# The OLIF working corridor based on magnetic resonance imaging: a retrospective research

**DOI:** 10.1186/s13018-020-01654-1

**Published:** 2020-04-15

**Authors:** Zhe Wang, Lei Liu, Xiang-he Xu, Ming-de Cao, Hai Lu, Kui-bo Zhang

**Affiliations:** 1 Department of Spine Surgery, The Fifth Affiliated Hospital, Sun Yat-sen University, Zhuhai, China; 2 Department of Orthopaedics, The Fifth Affiliated Hospital, Sun Yat-sen University, Zhuhai, China

**Keywords:** OLIF, Complication, Vascular injury, Magnetic resonance imaging, Working corridor

## Abstract

**Objective:**

To provide an anatomical basis for the development of oblique lumbar interbody fusion (OLIF) in Chinese patients.

**Methods:**

Between November 2018 and June 2019, 300 patients’ lumbar MRI data were reviewed. According to the Moro system and zone method described by us, the axial view was vertically divided into 6 zones (A, I II, III, IV, P) and was horizontally divided into 4 zones (R, a, b, c, L). The locations of left psoas muscle and the major artery at L2/3, L3/4, and L4/5 levels were evaluated by the grid system. The aortic bifurcation segments will also be evaluated at the level of the vertebral body or the disc.

**Results:**

At the L2/3 level, left psoas muscle and the major artery in zone Ib were found in 28.0% of subjects, in zone IIb in 20.3%, and in zone Ic in 20.0%; at the L3/4 level, in zone Ab in 20.7% of subjects, in zone Ac in 26.0%, and in zone Ic in 11.0%; and at the L4/5 level, areas in zone Ab in 31.0% of subjects, in zone Ac in 26.0%, and in zone Ib in 11.7%. The aortic bifurcation segments were mainly at the L4 level. The zone of the left psoas muscle at all levels, the zone of the major artery at L4/5 level, and the zone of the aortic bifurcation segments had significant correlation with gender difference (*P* < 0.05).

**Conclusion:**

The left-sided OLIF at L2–L5 disc levels can be a feasible type of surgery for lumbar interbody fusion in the majority of Chinese patients. Before the operation, in order to screen out the appropriate surgical approach, routine lumbar magnetic resonance imaging is recommended to analyze the patient’s local anatomical features.

## Introduction

In 1944, it was the first time that Briggs and Milligan introduced lumbar interbody fusion, which exerted far-reaching impact on the development spinal surgery [1]. Today, this type of classic surgical treatment has been widely used in a variety of lumbar degenerative diseases, which consist of lumbar intervertebral disc herniation, lumbar spinal stenosis, degenerative lumbar scoliosis, and degenerative spondylolisthesis [2]; however, early lumbar interbody fusion approaches mainly consist of posterior lumbar interbody fusion and transforaminal lumbar interbody fusion that would cause severe damage to the structures on the posterior side of the lumbar spine [3, 4]. In these years, with unremitting pursuit of minimally invasive surgery technology and continuous improvement of surgical instruments, these new minimally invasive technologies primarily include anterior lumbar interbody fusion (ALIF), extreme lumbar interbody fusion (XLIF), and a prepsoas or anterior to the psoas oblique lumbar interbody fusion (OLIF). The advantage of ALIF is to maintain the integrity of the posterior anatomy and to correct the sagittal deformity [5, 6], but it may lead to injury of the large bowel, vessel, and retrograde ejaculation [7, 8]. The merits of XLIF mainly lie in shorter operation time and less bleeding volume, larger cage placement, decreased tissue trauma, and high fusion rate [9, 10]; however, its demerits include neural, visceral injuries, and approach-related psoas muscle damage [11].

OLIF has emerged in recent years, which was firstly described by Silvestre et al. [12]. Unlike the transpsoas approach of the XLIF, the OLIF takes full advantage of the natural cavity between the psoas muscle and the blood vessels to get access to the intervertebral discs [13]. Therefore, the injury to lumbar plexus or femoral nerve could be prevented [14]. This type of surgery is increasingly popular with spine surgeons. Although by means of the natural corridor, some complications such as vascular injury should be paid much attention [12]. Some previous studies have reported the incidence of vascular damage, between about 0.8% and 10%. Ohtori et al. [14] reported 1 case of segmental artery injury in their 35 subjects cohort study. Silvestre et al. [12] reported a vascular damage rate of 1.7% in 179 subjects. Shunsuke Fujibayashi et al. [15] reported 1 case of major vascular injury and 7 cases of segmental artery injury in 992 patients. Jiaqi Li et al. [16] reported 3 cases of vascular injury in 30 patients. Despite the above research that the probability of major vascular (abdominal aorta or iliolumbar vessel) injury is very small, this type of injury is almost fatal.

As for a spine surgeon, clear understanding of the anatomical features and regularities of the surgical corridor may be an effective measurement to avoid such intraoperative complications. The purpose of this study was to explore the characteristics of the left-sided oblique channel in the community. We retrospectively analyzed the physiological and anatomical data of lumbar MRI in 300 subjects, that is, the relationship between the left psoas muscle and the abdominal aorta or the left common iliac artery, which provided an anatomical basis for the development of OLIF in Chinese patients.

## Materials and methods

We retrospectively reviewed imaging data from 300 patients (167 males and 133 females) who underwent 1.5 T MRI of the lumbar spine between October 2018 and June 2019. These patients ranged in age from 18 to 89 years, with an average age of 47.3 years. All subjects had clear axial and sagittal MRI scans. Patients who had abdominal vascular abnormalities or diseases, spinal deformity, lumbar fracture, or a surgical history on lumbar or retroperitoneum were excluded.

**Fig. 1:**
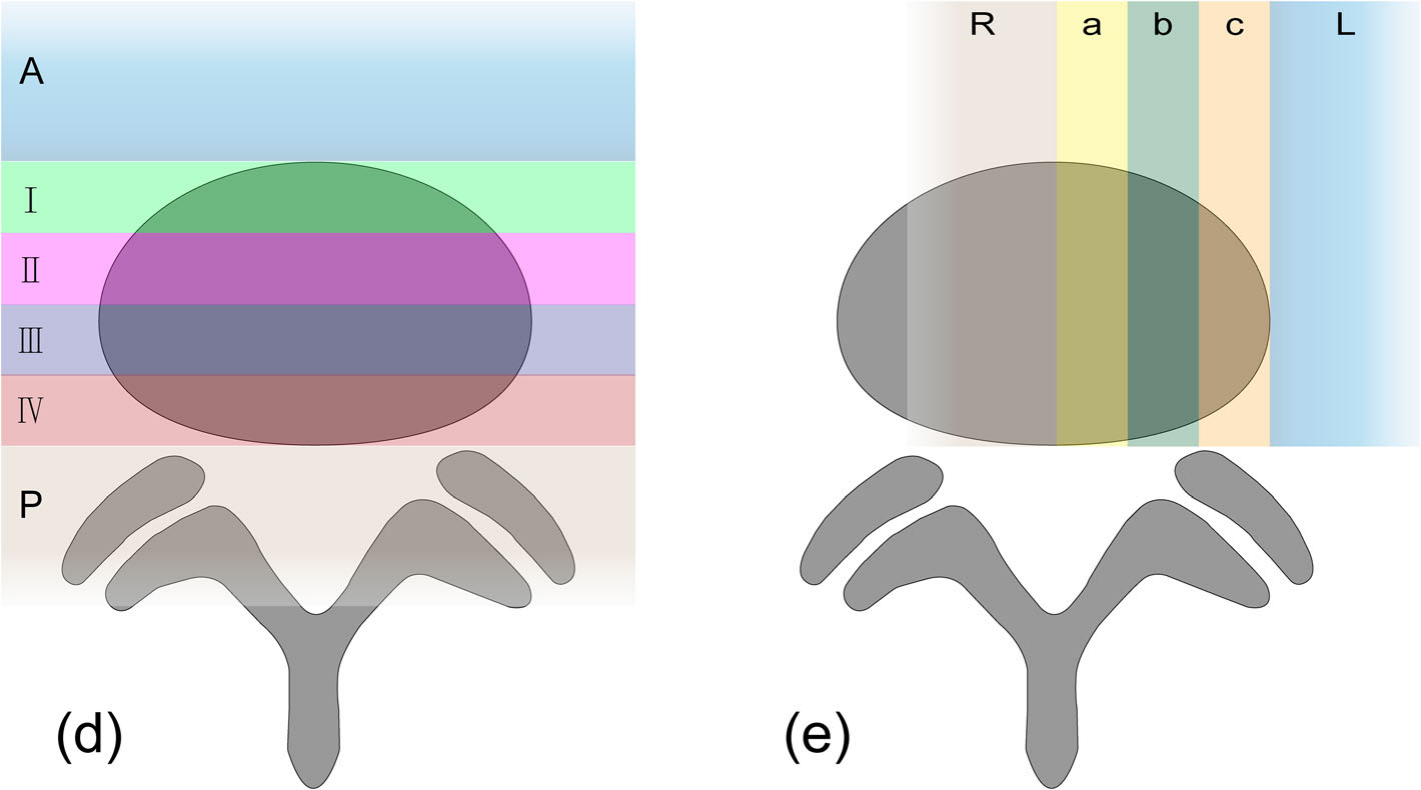
**d** Represents the location of psoas muscle at every disc levels. The axial plane of 3 intervertebral discs was divided into 6 zones according to the method by Moro et al. [17], and the front tangential lines of the left psoas muscle were evaluated. The area between the anterior and posterior edge of the intervertebral disc was divided into zones I, II, III, and IV. The area anterior to the anterior edge and the area posterior to the posterior edge of the intervertebral disc were defined as zone A and zone P respectively. **e** Represents the location of major artery at every disc levels and the left part the axial plane of 3 intervertebral discs was divided into 5 zones, and the right tangential lines of the major artery were measured. The area between the median line(passing through the center of the disc and the spinous process)and the left edge of the intervertebral disc was divided into zones a, b, and c. The area right to the median line and the area left to the left edge of the intervertebral disc were defined as zone R and zone L respectively

The most caudal mobile lumbar spine would be described as L5 in this study. According to the left-sided OLIF approach, we defined the section from the abdominal aorta to the left common iliac artery as the major artery. Axial and sagittal planes from Centricity radiology information system (Neursoft Company, Liaoning Province, CHN) were used to locate the L2-3, L3-4, and L4-5 intervertebral discs. The related parameters of major artery and left psoas major were analyzed and recorded in an axial plane. When analyzing the left psoas muscle, the axial plane of 3 intervertebral discs was divided into 6 zones according to the method by Moro et al. [17], and the front tangential lines of the left psoas muscle were evaluated. The area between the anterior and posterior edge of the intervertebral disc was divided into zones I, II, III, and IV. The area anterior to the anterior edge and the area posterior to the posterior edge of the intervertebral disc were defined as zone A and zone P respectively (Fig. 1d). If the front tangential line of the left psoas muscle was on the borderline, it was considered to be in the more frontal zone. As for the major artery, the axial plane of 3 intervertebral discs was divided into 5 zones, and the left tangential lines of the major artery were measured. The area between the median line (passing through the center of the disc and the spinous process) and the left edge of the intervertebral disc were divided into zones a, b, and c. The area right to the median line and the area left to the left edge of the intervertebral disc were defined as zone R and zone L respectively (Fig. 1e). The aortic bifurcation segments will also be evaluated at the level of the vertebral body or the disc. The Pearson Contingency Coefficient in SPSS Statistics Version 25.0 was used to analyze the correlation between sex difference and the zone of the front tangential line of the left psoas muscle or the zone of the left tangential lines of the major artery with a *p* value of 0.05. And Pearson Contingency Coefficient also was used to analyze the correlation between gender difference and bifurcation segments of aorta with a *p* value of 0.05.

## Results

### At the L2 to L3 levels

**Fig. 2:**
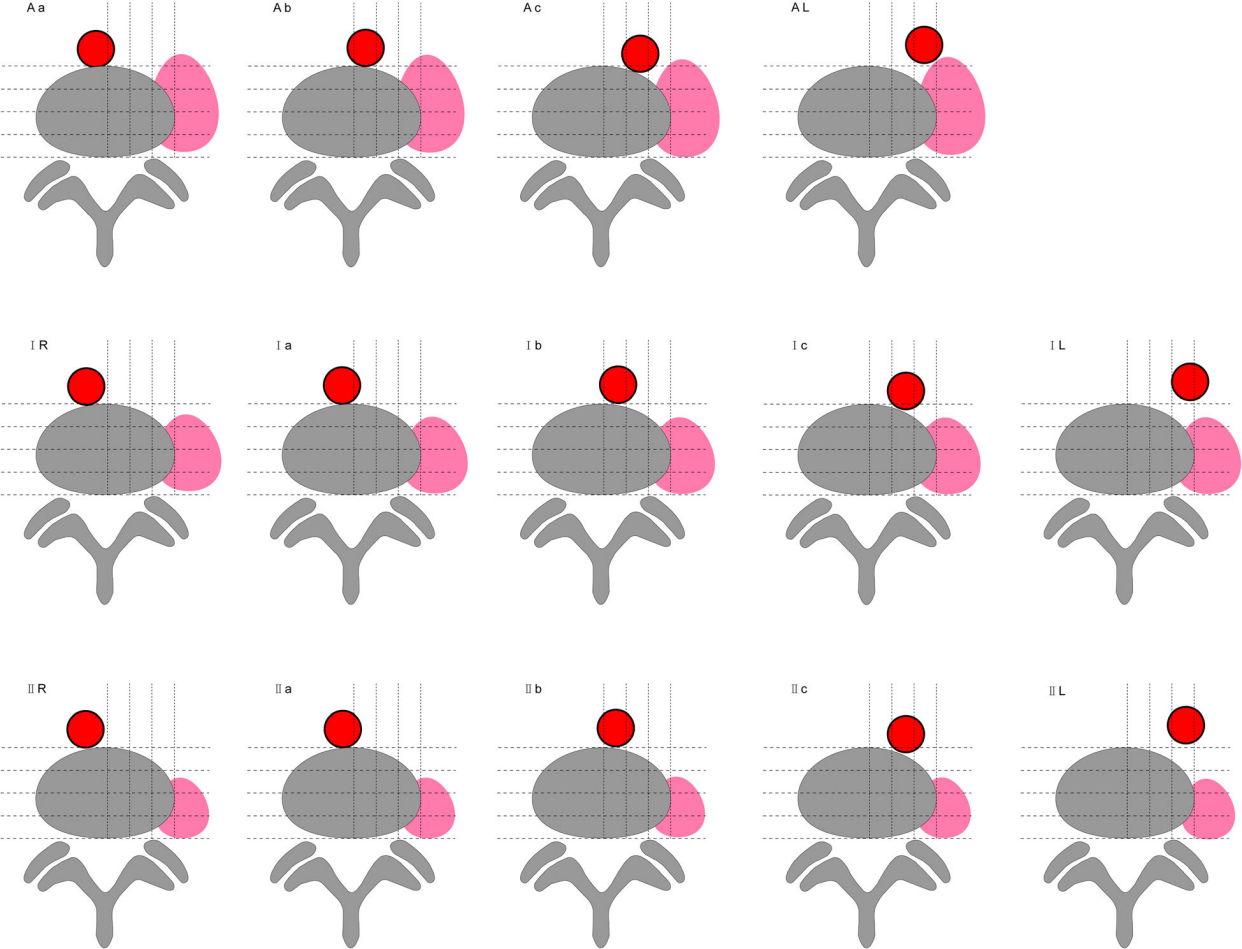
According to the existing situation of 300 subjects, all the combinations of psoas muscle and major blood vessel position are listed in this figure

**Fig. 3:**
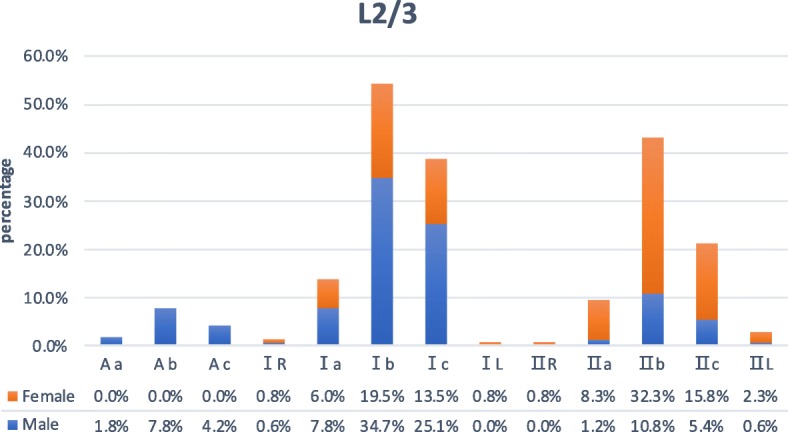
At the L2/3 level, there are ratios of locations where the front tangential line of the left psoas muscle and the left tangential line of the major artery in male and female subjects respectively

Areas where the front tangential line of the left psoas muscle and the left tangential line of the major artery are located in zone Aa 3 subjects (1.0%), in zone Ab in 13 (4.3%), in zone Ac in 7 (2.3%), in zone IR in 2 (0.7%), in zone Ia in 21 (7.0%), in zone Ib in 84 (28.0%), in zone Ic in 60 (20.0%), in zone IL in 1 (0.3%), in zone IIR in 1 (0.3%), in zone IIa in 13 (4.3%), in zone IIb in 61 (20.3%), in zone IIc in 30 (10.0%), and in zone IIL in 4 (1.3%) (Figs. 2 and 3). Sex difference had a significant correlation with the zone of the front tangential line of the left psoas muscle (*P* < 0.05), but sex difference had no significant correlation with the zone of the left tangential line of the major artery (*P* > 0.05).

### At the L3 to L4 levels

**Fig. 4:**
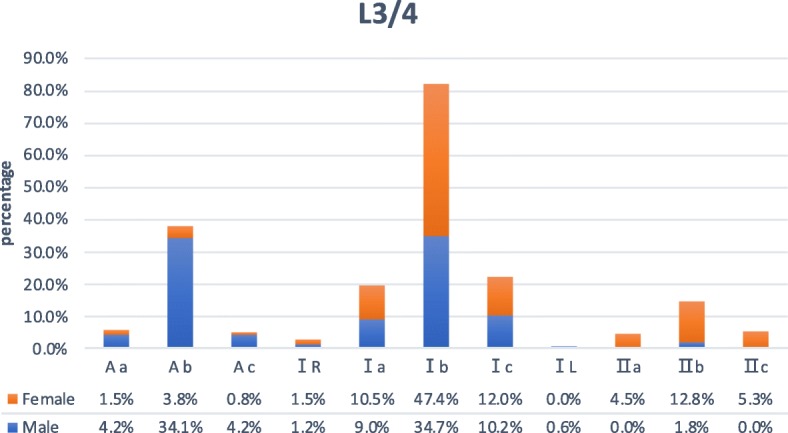
At the L3/4 level, there are ratios of locations where the front tangential line of the left psoas muscle and the left tangential line of the major artery in male and female subjects respectively

At this level, areas where the front tangential line of the left psoas muscle and left tangential line of the major artery are located in zone Aa 9 subjects (3.0%), in zone Ab in 62 (20.7%), in zone Ac in 8 (2.7%), in zone IR in 4 (1.3%), in zoneIa in 29 (9.7%), in zone Ib in 121 (40.3%), in zone Ic in 33 (11.0%), in zone IL in 1 (0.3%), in zone IIa in 6 (2.0%), in zone IIb in 20 (6.7%), and in zone IIc in 7 (2.3%)( Figs. 2 and 4). Sex difference had a significant correlation with the zone of the front tangential line of the left psoas muscle (*P* < 0.05), but sex difference had no significant correlation with the zone of the left tangential line of the major artery (*P* > 0.05).

### At the L4 to L5 levels

**Fig. 5:**
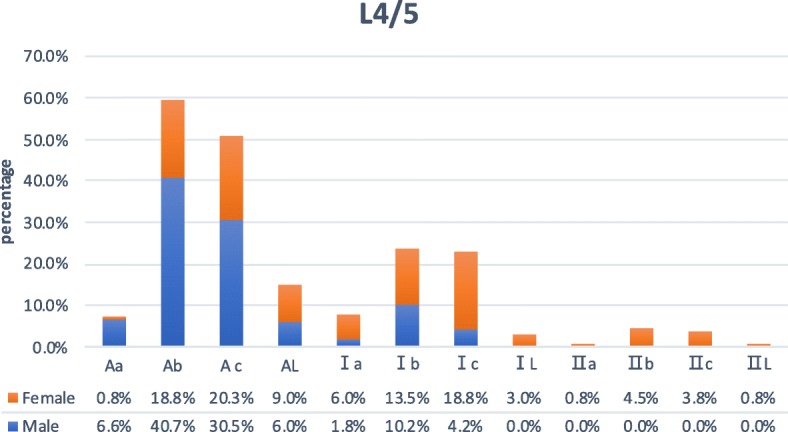
At the L4/5 level, there are ratios of locations where the front tangential line of the left psoas muscle and the left tangential line of the major artery in male and female subjects respectively

At this level, areas where the front tangential line of the left psoas muscle and the left tangential line of the major artery are located in zone Aa 12 subjects (4.0%), in zone Ab in 93 (31.0%), in zone Ac in 78 (26.0%), in zone AL in 22 (7.3%), in zone Ia in 11 (3.7%), in zone Ib in 35 (11.7%), in zone Ic in 32 (10.7%), in zone IL in 4 (1.3%), in zone IIa in 1 (0.3%), in zone IIb in 6 (2.0%), in zone IIc in 5 (1.7%), and in zone IIL in 1 (0.3%) (Figs. 2 and 5). Sex difference had a significant correlation with the zone of the front tangential line of the left psoas muscle (*P* < 0.05), and sex difference also had a significant correlation with the zone of the left tangential line of the major artery (*P* < 0.05).

Among the 300 subjects, the aortic bifurcation segments of both men (122, 73.1%) and women (99, 74.0%) were mainly at the L4 level. And gender difference also had a significant correlation with the bifurcation segments of aorta (*P* < 0.05).

## Discussion

Lumbar interbody fusion is a widely applied surgery that places of an implant (cage, spacer or structural graft) in the area between the upper and lower vertebral bodies after discectomy and endplate preparation [18]. OLIF, as a minimally invasive approach, unlike ALIF and XLIF, has its own unique surgical procedure. With the patient in right lateral decubitus position, a skin incision is performed on the basis of the patient’s surgical segment and preoperative fluoroscopy. Bluntly separating the external oblique muscle, obliquus internus abdominis muscle and transversus abdominis muscle, and then into the retroperitoneal space continues blunt division until the area of the left psoas muscle and aorta is reached and what we refer to as the working corridor is the natural space between left psoas and aorta [4]. Owing to this natural space, OLIF is different from XLIF, and intraoperative neuromonitoring is not required [18]. However, any kind of surgical operation inevitably has its corresponding risks. In recent years, more and more papers have reported cases of vascular injury related to OLIF operation. In 2012, Clément Silvestre et al. [12] reported 3 patients occurred with vascular injury (iliac or iliolumbar vein). In 2017, Kamal R.M. et al. [19] reported four cases of vascular injury. In 2017, Shunsuke Fujibayashi et al. [15] reported one major vascular injury. In 2019, Jiaqi Li et al. [16] reported 3 cases of vascular injury. Therefore, for the spine surgeon, careful operation during surgery and mastery of the anatomy are necessary. This study retrospectively analyzed the lumbar magnetic resonance axial scan of 300 patients to find the anatomic laws of left-sided OLIF working channel, which refers to the left psoas muscle and abdominal aorta or left common iliac artery.

According to the results of this study, at the L2/3 level, the proportion of male (82%) psoas muscle in zone A and zone I is relatively higher than that of women (39.5%), while in zone II, women (60.5%) are higher than men (18%). The majority of the subject’s major artery are distributed in zone a, zone b, and zone c (97.3%), and only a few are located in zone R and zone L (2.6%); At the L3/4 level, the proportion of male (42.5%) psoas muscle in zone A is relatively higher than that of women (6.1%), while in zone I and zone II, women (93.9%) are relatively higher than men (57.5%). The majority of the subject’s major artery are distributed in zone a, zone b, and zone c (98.4%), and only a few are located in zone R and zone L (1.6%); At the L4/5 level, the proportion of male (83.8%) psoas muscle in zone A is relatively higher than that of women (48.9%), while in zone I and zone II, women (51.1%) are relatively higher than men (16.2%). The majority of the subject’s major artery are distributed in zone b and zone c (83.0%), and only a few are located in zone R and zone L (17%). At L2/3, L3/4, and L4/5 levels, sex difference had a significant correlation with the zone of the front tangential line of the left psoas muscle. At the L2/3 level, the area of the psoas muscle of female subjects is only distributed in zone I and zone II, and at the L4/5 level, the area of the psoas muscle of male subjects is only distributed in zone A and zone I. One of the reasons may be that the psoas muscle has a larger volume in males than females [20]. Of the 300 subjects, none of the one’s psoas muscle areas were located in zone III, zone IV, and zone P, which is probably the normal anatomical feature of the major psoas muscle. The zone AL was only seen in L4/5, 10 cases (6.0%) in men and 12 cases (9.0%) in women, with a total of 22 cases (7.3%). In these cases, due to the narrow surgical working corridor and high risk of major vascular injury, it is not recommended to choose OLIF. XLIF would be the better choice, since XLIF is a lateral retroperitoneal transpsoas approach, which involves dissection traction of the psoas muscle, and the concentration of lumbar plexus was located mostly on the posterior substance of the major psoas [21]. Subjects in zone AL, in these cases, their left psoas muscle and the aorta were almost next to each other, and the OLIF left-sided approach does not seem to be a wise choice. And transpsoas approach or anterior approach would be more relatively suitable. On these three levels, there were a few subjects with the zone IL and Ac. These two kinds of interspace between left psoas muscle and major artery seemingly offer a compact and severe OLIF working corridor, especially for young surgeons who are in the early stages of the learning curve of OLIF approach [16]. On the contrary, patients in zone IIR and IIa provide a wider natural working corridor and theoretically decrease the rate of approach-related intraoperative complication [12, 18, 22]. Only at the L4 to L5 level, gender difference had a significant correlation with the zone of the left tangential line of the major artery. It could be affected by aortic bifurcation partly.

At present, there are a few researches to study the working corridor of OLIF. Davis et al. [23] investigated the left-sided oblique channel in the L2–S1 disc planes in 20 cadaveric specimens while maintaining the same surgical right lateral decubitus position as OLIF, and the conclusion was that the largest and smallest corridor located in the L3-4 level and the L4-5 level respectively. Diana et al. [24] analyzed 100 MR images of the lumbar spine to investigate the left-sided retroperitoneal oblique corridor of L2–S1 and got a conclusion of (1) 90% of the subjects had oblique access to each of the L2–L5 intervertebral discs and (2) only 69% had clear access to the L5–S1 intervertebral discs. Liu et al. [25] analyzed 60 adults through abdominal computed tomography angiography and computed tomography of T12-S1 vertebral with three-dimensional reconstruction, then they deemed that the working corridor of each level related to OLIF has its own anatomical peculiarities, and not all levels are suitable for left-sided OLIF. And Chen et al. [13] studied 400 subjects by lumbar spine MRI and concluded that OLIF in a left-sided approach can be suitable for the most Chinese patients from L2 to S1 segment; meanwhile, the more surgical space of the OLIF is seen in older female patients.

This study aims to provide applied anatomical evidence of assessment of left-sided OLIF before the operation, according to the system proposed by Moro et al [17].; meanwhile, we have made improvements and innovations, providing a simple and feasible anatomical partition suitable for the left psoas muscle and the major artery. For whether to choose the left-sided or right-sided approach, Jia Xi Julian Li et al. [26] analyzed the size of the OLIF working corridor both on the left and right side by MRI, and found that the size of the OLIF working corridor on the left side tends to be larger than it on the right side. Therefore, this study only focused on analyzing the left-sided OLIF approach. Although there was a small number of the successful case of OLIF surgery on L5/S1 leve l[27], OLIF approach is not likely to be preferred a suitable choice on an account of risks associated with manipulation of the vessels and the presence of the iliac wing. According to the research by Jia Xi Julian Li et al. [26], the left-sided oblique access might be blocked by the left kidney (22.5%) and accompanying renal vasculature (52%). Hence, L1/L2 and L5/S1disc levels are not included in the study. Thanks to the advantage of MRI for soft tissue imaging is clearer than CT, we retrospectively analyzed of lumbar MRI scans from 300 patients randomly who consist of the young and old, male, and female. Interestingly, however, Zhang et al. [28] conducted a research on whether changes in the patient’s position would affect the size of the surgical corridor by MRI. They found a decrease in the oblique corridor size from the right lateral decubitus position to the supine position on MRI, and pointed out emphatically that using MRI scans in the supine position to analyze the morphometric features of oblique corridor might not be accurate. The authors thought the main reason was deformation of left psoas muscle caused by gravity, and the front edge of the relaxed muscle is closer to the major artery [28]. But, we prefer to support the thought of Jia Xi Julian Li et al. [26], that is, we would inevitably retract the psoas muscle to some extent during intraoperative manipulation; if we retract the part of the muscle that has moved forward due to gravity back to the original position, it would not cause severe damage to the psoas major or nerves. Therefore, lumbar MRI in the supine position can still be used as an effective examination for the preoperative evaluation of OLIF. We hope to offer spine surgeons the reliable population anatomical data and a convenient and feasible imaging analysis method.

The present research has some limitations. First, there may be some degree of human error in the measurement of each parameter. Second, the current study did not involve analysis of the patient’s lumbar spine deformities, although these patients in clinical practice remain a challenging difficulty. Finally, the current research is just a morphometric preoperative imaging study with respect to the left-sided OLIF approach. Due to the lack of clinical data on various positional relationships between the major artery and psoas muscle, it is not sufficient to demonstrate safety and reliability of the OLIF approach in the specific location of the muscle and blood vessel. And this may be our future research objective and contents.

## Conclusions

The left-sided OLIF at L2–L5 disc levels can be a feasible type of surgery for lumbar interbody fusion in the majority of Chinese patients. Before the operation, in order to screen out the appropriate surgical approach, routine lumbar magnetic resonance imaging is recommended to analyze the patient’s local anatomical features.

## Data Availability

The patients’ data were collected in the Fifth Affiliated Hospital, Sun Yat-sen University. The datasets used and/or analyzed during the current study are available from the corresponding author upon reasonable request.

## Abbreviations

ALIF: Anterior lumbar interbody fusion
MRI: Magnetic resonance imaging
OLIF: Oblique lumbar interbody fusion
XLIF: Extreme lumbar interbody fusion

## References

[1] Kaiser MG, Eck JC, Groff MW, Watters WC, Dailey AT, Resnick DK, Choudhri TF, Sharan A, Wang JC, Mummaneni PV (2014). Guideline update for the performance of fusion procedures for degenerative disease of the lumbar spine. Part 1: introduction and methodology. J Neurosurg Spine.

[2] Mummaneni PV, Dhall SS, Eck JC, Groff MW, Ghogawala Z, Watters WC, Dailey AT, Resnick DK, Choudhri TF, Sharan A (2014). Guideline update for the performance of fusion procedures for degenerative disease of the lumbar spine. Part 11: interbody techniques for lumbar fusion. J Neurosurg Spine.

[3] DiPaola CP, Molinari RW (2008). Posterior lumbar interbody fusion. J Am Acad Orthop Surg.

[4] Xu DS, Walker CT, Godzik J, Turner JD, Smith W, Uribe JS (2018). Minimally invasive anterior, lateral, and oblique lumbar interbody fusion: a literature review. Ann Transl Med.

[5] Regan JJ, Guyer RD (1997). Endoscopic techniques in spinal surgery. Clin Orthop Relat Res.

[6] Regan JJ, Yuan H, McAfee PC (1999). Laparoscopic fusion of the lumbar spine: minimally invasive spine surgery A prospective multicenter study evaluating open and laparoscopic lumbar fusion. Spine (Phila Pa 1976).

[7] Rajaraman V, Vingan R, Roth P, Heary RF, Conklin L, Jacobs GB (1999). Visceral and vascular complications resulting from anterior lumbar interbody fusion. J Neurosurg.

[8] Sasso RC, Kenneth Burkus J, LeHuec JC (2003). Retrograde ejaculation after anterior lumbar interbody fusion: transperitoneal versus retroperitoneal exposure. Spine (Phila Pa 1976).

[9] Ozgur BM, Aryan HE, Pimenta L, Taylor WR (2006). Extreme lateral interbody fusion (XLIF): a novel surgical technique for anterior lumbar interbody fusion. Spine J.

[10] Dangelmajer S, Zadnik PL, Rodriguez ST, Gokaslan ZL, Sciubba DM (2014). Minimally invasive spine surgery for adult degenerative lumbar scoliosis. Neurosurg Focus.

[11] Sakai T, Tezuka F, Wada K, Abe M, Yamashita K, Takata Y, Higashino K, Sairyo K (2016). Risk Management for avoidance of major vascular injury due to lateral transpsoas approach. Spine (Phila Pa 1976).

[12] Silvestre C, Mac-Thiong JM, Hilmi R, Roussouly P (2012). Complications and morbidities of mini-open anterior retroperitoneal lumbar interbody fusion: oblique lumbar interbody fusion in 179 patients. Asian Spine J.

[13] Chen X, Chen J, Zhang F. Imaging anatomic research of oblique lumbar interbody fusion in a Chinese population based on magnetic resonance. World Neurosurg. 2019. 10.1016/j.wneu.2019.03.244 31035020

[14] Ohtori S, Orita S, Yamauchi K, Eguchi Y, Ochiai N, Kishida S, Kuniyoshi K, Aoki Y, Nakamura J, Ishikawa T (2015). Mini-open anterior retroperitoneal lumbar interbody fusion: oblique lateral interbody fusion for lumbar spinal degeneration disease. Yonsei Med J.

[15] Fujibayashi S, Kawakami N, Asazuma T, Ito M, Mizutani J, Nagashima H, Nakamura M, Sairyo K, Takemasa R, Iwasaki M (2017). Complications associated with lateral interbody fusion: nationwide survey of 2998 cases during the first 2 years of its use in Japan. Spine.

[16] Li J, Wang X, Sun Y, Zhang F, Gao Y, Li Z, Ding W, Shen Y, Zhang W: Safety Analysis of Two anterior lateral lumbar interbody fusions at the initial stage of learning curve. (1878-8769 (Electronic)). 10.1016/j.wneu.2019.03.294 30959256

[17] Moro T, Kikuchi S, Konno S, Yaginuma H: An anatomic study of the lumbar plexus with respect to retroperitoneal endoscopic surgery. Spine (Phila Pa 1976) 2003, 28(5):423-428; discussion 427-428. 10.1097/01.BRS.0000049226.87064.3B 12616150

[18] Mobbs RJ, Phan K, Malham G, Seex K, Rao PJ (2015). Lumbar interbody fusion: techniques, indications and comparison of interbody fusion options including PLIF, TLIF, MI-TLIF, OLIF/ATP. LLIF and ALIF. J Spine Surg.

[19] Woods KRM, Billys JB, Hynes RA (2017). Technical description of oblique lateral interbody fusion at L1–L5 (OLIF25) and at L5–S1 (OLIF51) and evaluation of complication and fusion rates. The Spine Journal.

[20] Urrutia JA-Ohoo, Besa P, Lobos D, Campos M, Arrieta C, Andia M, Uribe S: Lumbar paraspinal muscle fat infiltration is independently associated with sex, age, and inter-vertebral disc degeneration in symptomatic patients. (1432-2161 (Electronic)). 10.1007/s00256-018-2880-1 29379999

[21] Tubbs RI, Gabel B, Jeyamohan S, Moisi M, Chapman JR, Hanscom RD, Loukas M, Oskouian RJ, Tubbs RS (2017). Relationship of the lumbar plexus branches to the lumbar spine: anatomical study with application to lateral approaches. Spine J.

[22] Phan K, Maharaj M, Assem Y, Mobbs RJ: Review of early clinical results and complications associated with oblique lumbar interbody fusion (OLIF). J Clin Neurosci 2016, 31(1532-2653 (Electronic)):23-29. 10.1016/j.jocn.2016.02.030 27349468

[23] Davis TT, Hynes RA, Fung DA, Spann SW, MacMillan M, Kwon B, Liu J, Acosta F, Drochner TE (2014). Retroperitoneal oblique corridor to the L2-S1 intervertebral discs in the lateral position: an anatomic study. J Neurosurg Spine.

[24] Molinares DM, Davis TT, Fung DA (2016). Retroperitoneal oblique corridor to the L2-S1 intervertebral discs: an MRI study. J Neurosurg Spine.

[25] Liu L, Liang Y, Zhang H, Wang H, Guo C, Pu X, Zhang C, Wang L, Wang J, Lv Y (2016). Imaging anatomical research on the operative windows of oblique lumbar interbody fusion. PLoS One.

[26] Julian Li JX, Mobbs RJ, Phan K: Morphometric MRI imaging study of the corridor for the oblique lumbar interbody fusion technique at L1-L5. (1878-8769 (Electronic)). 10.1016/j.wneu.2017.12.136 29294391

[27] Kanno K, Ohtori S, Orita S, Yamauchi K, Eguchi Y, Aoki Y, Nakamura J, Miyagi M, Suzuki M, Kubota G et al: Miniopen oblique lateral L5-s1 interbody fusion: a report of 2 cases. Case Rep Orthop 2014, 2014(2090-6749 (Print)):603531. 10.1155/2014/603531 PMC4221972 25400963

[28] Zhang F, Xu H, Yin B, Tao H, Yang S, Sun C, Wang Y, Yin J, Shao M, Wang H et al: Does right lateral decubitus position change retroperitoneal oblique corridor? A radiographic evaluation from L1 to L5. (1432-0932 (Electronic)). 10.1007/s00586-016-4645-7 27272493

